# Surgery of the Antrum

**Published:** 1892-08

**Authors:** G. S. Junkerman

**Affiliations:** Cincinnati


ARTICLE VIII.

### SURGERY OF THE ANTRUM.

The antrum is only one of the air cells of the skull, and since it is of importance as the largest of these, it is known by the dignified title of the "Antrum of Highmore." It, like other air cells of the skull, is lined by the Schneiderian membrane. It is of significance to the oral surgeon because it comes within his jurisdiction to treat its diseases, and it is the only air cavity that bears any relationship with the teeth. The antrum is located mainly within the superior maxillary bone, but its walls are completed only by the addition of the inferior turbinated, lachrymal and palate bones, which bones are interested in forming most of its nasal wall. It is triangular in shape, with its apex pointing to the canine fossa. The base looks backward toward the palate. The other walls look respectively toward the nasal fossa, the orbit and the palatine process of the superior maxillary bone. If this cavity has any function I think it differs somewhat from the function of the remaining air cells of the head, viz., to warm the air before it passes to the lungs. To retain the contour of the face nature has combined in this bone, by the formation of this air cavity, both lightness of weight and extent of surface. She has furnished an ample frame for the face and yet retained the remainder of weight in the poise of of the head. If the cavity were solid bone tissue, it would make a remarkable difference in the weight of the head. There is another feature of importance which is the better maintenance of blood supply to the face, and of direct importance to the dental surgeon, in that by the presence of this cavity a freer supply of blood and nerve force to the molars and pre-molars is maintained. All diseases of this cavity would be treated surgically. Yet we would not wish to magnify the importance of the diseases connected with it. It is subject to about the same diseases that may affect any other mucous membrane; add to this the fact of its being a cavity of some obscurity, and rather restricted walls, and we have in view all the obstacles with which we have to contend in diagnosis and treatment. Any antral disease attains importance, not as regards the antrum itself, but as the progress of the disease affects the surrounding tissues. In most cases while the disease is confined to its own walls there is little or no inconvenience to the patient; but should it press upward, the orbital cavity is invaded. If the disease should extend at the expense of the inner wall of the cavity, the nasal fossa would be invaded, and should the floor of the cavity give way, the mouth would be associated with the disease. A forward projection of the disease would involve the face. From this we find that the oral orbital and the nasal cavities are not only liable to be connected in disease with the antrum, but owing to the thinness of the partition walls they are easily invaded.

For purpose of diagnosis and treatment of any disease of the antrum, access to the cavity must be gained. There are three ways of entering the antrum. There are two ways practical to the oral surgeon, and both of these are by way of the mouth. These are by way of the canine fossa, which is a little depression just posterior to the root of the upper cuspid, and by way of the alveolar cavities of the bicuspids and molars. The third entrance is by way of the middle meatus of the nasal fossa. This, in reality, is the only entrance which in the recent state is open. The dental surgeon is not equipped with instruments for treatment through this opening, and had better restrict himself to the other means of egress. Entrance should be gained into the antrum by way of the canine fossa when the apex of the cavity can be definitely located. The disease may indicate the position of this opening, and under the circumstances you are justified in perforating with a steel instrument and enlarging the opening to a convenient size. This is especially indicated if the teeth are all present and found to be intact.

Engorgement of the antrum characterized by facial tumor is the indication for this plan of procedure. Where facial tumor is not present, there is no absolute rule by which you can reach the antrum through the canine fossa. The development of the antrum is so variable it cannot be perfectly determined just how far forward the cavity is developed. It may be as far forward as the cuspid or no farther than the first molar. There is little risk in forcing entrance through the palatine root of the second molar, and this means should always be resorted to if possible. Facial tumor and preservation of the teeth would indicate the canine fossa operation. The alveolar operations are preferable where the preservation of the teeth are not concerned. It would be of no purpose to enumerate the various diseases which may afflict the antrum and indicate their treatment. These points of information may be gained by consulting any chapter on mucous membranes and their diseases. With the antrum the diseases are complicated by their being confined to a cavity, Free and open drainage for the cavity is the first principle of treatment; antisepsis the other.

The operator is frequently brought in contact with the antrum accidentally in the process of extracting teeth. This may occur by forcing the root into the antral cavity, thereby fracturing the alveolar wall, or by tearing away part of the floor of the antrum with the root of the tooth. Either may produce a complication in the form of troublesome inflammation. The former mishap would necessitate the enlarging of the opening made, and a removal, if possible, of the foreign body. If this could not be accomplished, the opening should be enlarged.

There are, no doubt, many obscure pains, especially of a neuralgic nature about the head and face, that arise from antral trouble, and a little surgical interference will often relieve complication.

There are diseases of the antrum, that are brought by association with the other air cavities, amenable to treatment through the nasal opening of the antrum, but these are not in the fold of the oral surgeon.—G. S. JUNKERMAN, of Cincinnati, in *Items of Interest*.

